# A study on initiation of postpartum family planning in India based on NFHS-4: does urban poor differ significantly from rural?

**DOI:** 10.1186/s12905-022-02042-z

**Published:** 2022-11-24

**Authors:** Ujjaval Srivastava, Arvind Pandey, Pragya Singh, Kaushalendra Kumar Singh

**Affiliations:** 1 Indian Statistical Service, Government of India, Mumbai, India; 2 National Institute of Medical Statistics, ICMR, New Delhi, Ansari Nagar India; 3 Banaras Hindu University, Varanasi, India

**Keywords:** Postpartum family planning, NFHS-4, Urban poor, Maternal and child health, Survival analysis

## Abstract

**Objective:**

To explore the differentials of postpartum contraceptive adoption between rural and urban poor after adjusting for utilization of MCH services and other selected socioeconomic and demographic covariates.

**Methods:**

The data for this study is taken from the 4th round of NFHS survey conducted in India during 2015–16. The analysis is limited to 125,340 currently married women whose menses had returned at the time of survey. Discrete time complementary log–log multilevel model was applied.

**Results:**

The results clearly indicate that women from rural areas had a lower chance of early initiation of modern spacing methods after having recent birth as compare to that of Urban Poor and Urban non-poor areas. The contributions of several socioeconomic and demographic characteristics that were important for family planning practice were also highlighted in this study.

**Conclusion:**

There is an urgent need of designing an intervention that will result in effective delivery of services to achieve the greatest impact. Policy planners must focus on targeted interventions for family planning use in the postpartum period than simply focusing on family planning.

## Introduction

It is widely known that contraception would not only prevents births [1] but also contributes to reducing maternal mortality [2]. Irrespective of this evidence, the 2020 UN Population estimate reports that there are about 218 million women who want to prevent or delay pregnancies in developing countries, but they are not using any methods of contraception [3]. In 2018, a study in India reported that 11% of pregnancies had caused unintended births and 5% have caused miscarriages from those unintended births, and 33% of pregnancies have resulted in induced abortions [4]. According to World Family Highlights, more than one in ten married or in-union women have an unmet need for family planning worldwide; that is to say, they affirm that either they want to stop or delay their childbearing but are not using any contraceptive method to prevent their pregnancy. It is found that women in developing countries get to engage in sexual relationships without using contraception during their postpartum period [5], which results in an increased risk of infant and child mortality along with unwanted pregnancy and shorter birth intervals. Maternal mortality remains a significant public health challenge to the global population. Statistics indicate that an estimated 303,000 maternal death occurred worldwide, among which 99% of deaths were from developing countries should be at least 2–3 years of gap between births so that the women get time to restore their health and also to reduce infant and child mortality [6–8]. Postpartum Family Planning (PPFP) is defined as the start of the contraceptive method in the first twelve months after the delivery, which provides maternal and child benefits and birth spacing [9]. Worldwide most of communities practice abstinence after delivering a birth but with unknown or varying duration [10, 11]. Several studies like the one conducted in urban slums of Nairobi revealed the start of sexual relations prior to the resumption of menses [12, 13]. Similarly, the result of a study based in seventeen countries shows that women start to use contraceptive methods after their menstruation returns [13]. These studies depicts that these women are at high risk of unwanted birth as compared to those who have started using contraceptives before the start of menses. Breastfeeding cannot always be considered a safe alternative for amenorrhoeic women in place of contraceptive use to prevent them from unwanted pregnancy [14].

Unwanted pregnancy is linked to an increase in intentional abortion, preterm birth, low birth weight, and various other pregnancy-related problems [15–17]. It is widely accepted that an unintended pregnancy has a significant impact on the health of newborns [18]. It is stated that the majority of unintended pregnancies take place in developing nations, having a negative impact on the health, economic, and social development of communities [19].

The reports of the National Family Health Survey (NFHS-4) show the decline in modern contraceptive methods from 53.5% from 56.3% in NFHS-3 [20]. The low level of contraceptive use has been attributed, among other factors, to poor awareness of these methods among women [21–23], poor health infrastructure, and transportation facilities that hinder the approach to family planning services [8]. According to the NFHS-4 report, the high level of unmet need of family planning is 12.9% in India which makes the situation worse [20]. Thus, low uptake of contraceptives can be considered an essential reason for deteriorating maternal complications and promoting short birth intervals. Therefore, family planning needs to be promoted among women for better maternal and newborn health. There is ample literature [24–26] on postpartum contraceptives, but only a few studies that studies the duration of initiation of contraceptive use after giving birth. Also, there is a need to understand the various determinants of contraceptive methods used after the resumption of sexual relations following a delivery.

Today, as we know that India has entered the phase of rapid urbanization, with 34% of the population residing in urban areas [22]. This phase of increase in urbanization has led to an increase in urban poor residents, with about a quarter of 400 million of them being classified as urban poor. When we compare the health of urban poor women with rural, we find that they are equally undernourished or even worse than rural, but this disparity is masked by urban averages [27]. The health scenario of the urban poor is disadvantaged as the rural poor [28, 29]. It is a general belief that the urban population is at a high advantage in terms of health and economic conditions, but the proliferation of informal urban settlements (slums) that are increasingly absorbing the maximum of the urban population is at worse or at par with a rural population [30]. The characteristics of informal urban settlements include high population densities, widespread poverty and unemployment, criminality, insecure living conditions, and low-quality housing, poor hygiene, lack of access to water and sanitation facilities, as well as poor infrastructure, and exclusion from public and basic social services [30, 31]. The above-mentioned characteristics of urban area are associated with thedeterioration in urban health and social indicators, and especially the adverse outcomes in relation to Sexual and Reproductive Health Rights [32–35]. So, in order to further emphasize the situation of urban poor women, we have examined several objectives in this study.

## Objectives of the study

**Fig. 1:**
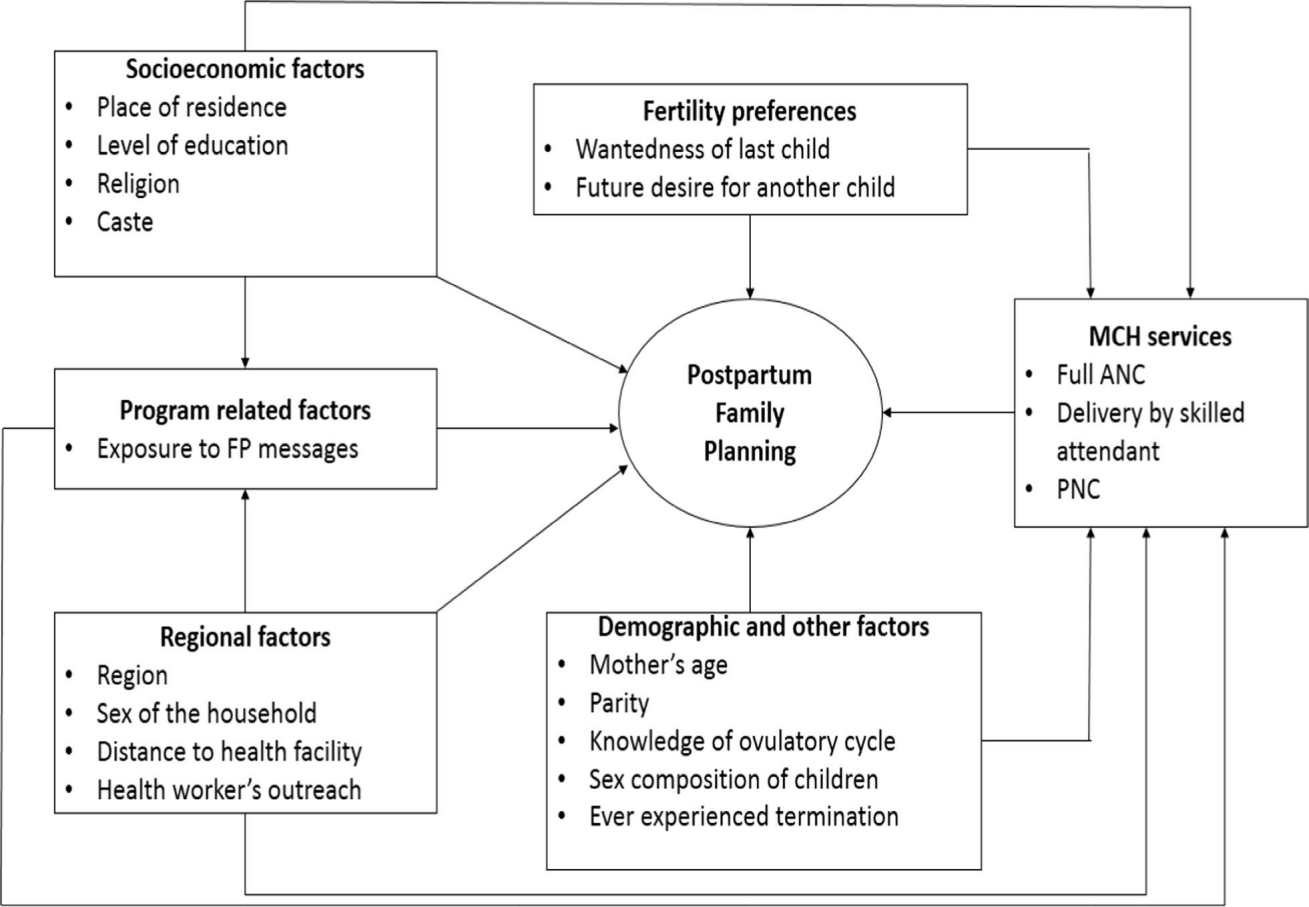
Conceptual framework of the study

The objectives of this study were as follows:

- To identify women initiating the use of any method of contraception within 12 months after childbirth.
- To analyze the differentials in the timing of initiation of modern spacing contraceptives within 12 months of childbirthby selected socioeconomic and demographic factors (Fig. 1).
- To examine the differential in the timing of initiation of modern spacing contraception within 12 months of childbirth between rural and urban poor women after adjusting for the selected covariates.
  Figure 1 graphically shows the conceptual framework of the study.

## Methodology

### Defining urban poverty

In the present study, urban sample was segregated into two parts: the poorest and poor quintiles representing theurbanpoor whereas rest represent the urban non-poor while rural sample was not disaggregated into two parts. In this way, the whole sample was divided into three parts; Rural, Urban Poor and Urban Non-Poor. To check the consistency of our definition, we compared our estimates of urban poor with that given by planning commission (2013) with Tendulkar methodology.

### Analytical sample

**Fig. 2:**
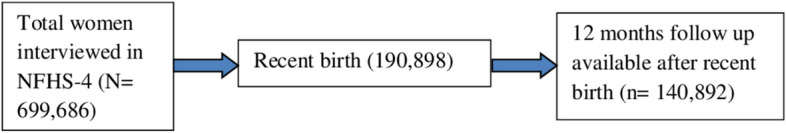
Study sample for Postpartum Family Planning (PPFP) analysis, India, NFHS-4

For fulfilling objectives of our study, we followed calendar history of every woman who gave birth during 13–60 months of calendar so that we can get 12 months follow up for each selected woman. In the present objective, left censored cases were events experienced by women before calendar start and were not the interest of study subject. Applying these selection criteria, the final study sample was 140,893 (weighted *n* = 136,953) women age15-49 (Fig. 2).

**Fig. 3:**
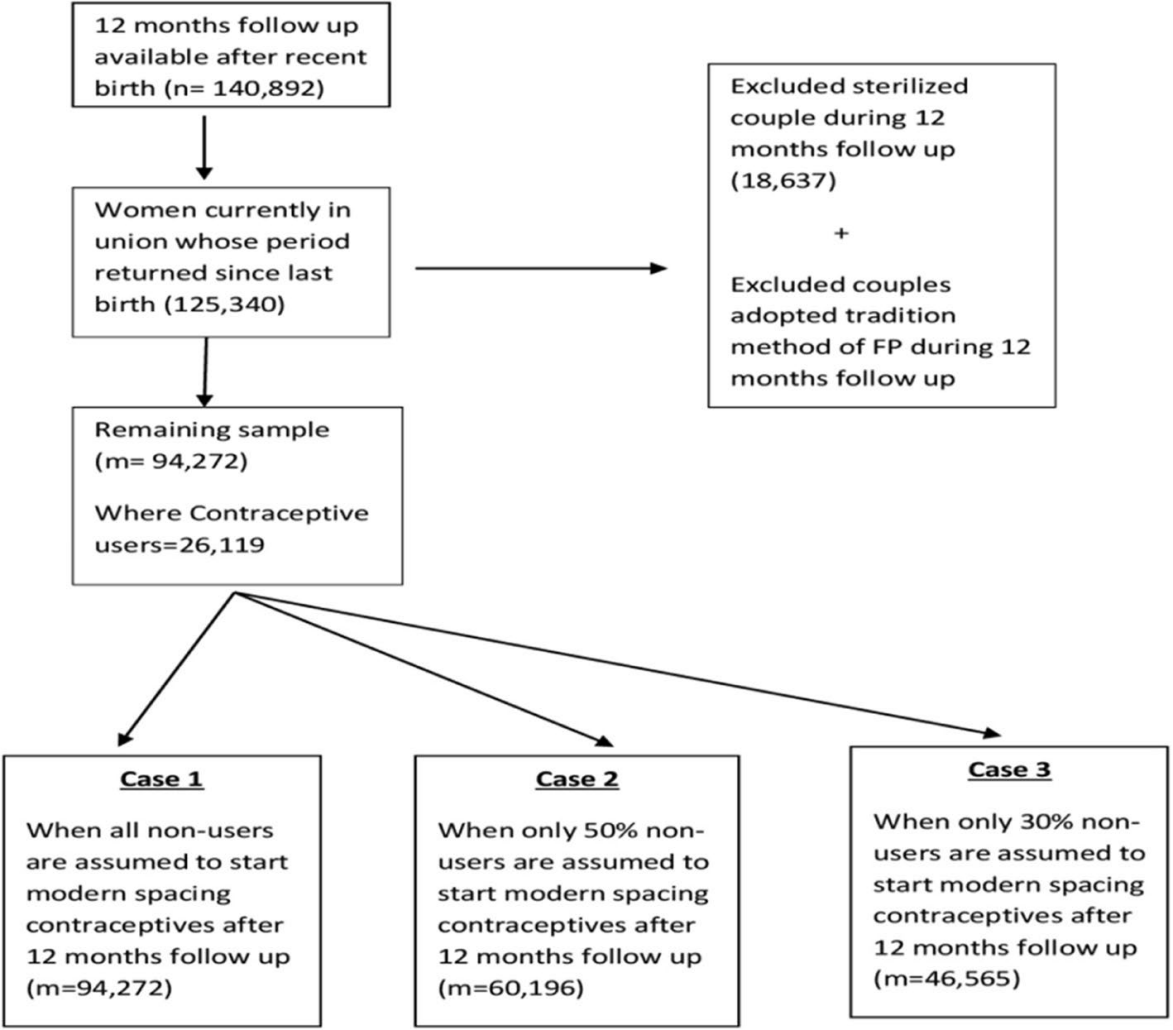
Study sample for time to adoption of spacing contraceptives analysis, India, NFHS-4

Previous research shows that FP use was more likely in the month following menses return [36]. Therefore, in order to analyze time to contraceptive adoption (only modern spacing methods considered here), analysis is limited to currently married women whose menses had returned at the time of survey (*n* = 125,340). The women who adopted limiting methods (*n* = 18,637) and traditional methods during 12 months of follow up period, were excluded for survival analysis. There final sample consists of 94, 272 eligible women for survival analysis (Fig. 3). After receiving permission from The Demographic and Health Surveys (DHS) Program the NFHS-4 data were downloaded from their website (http://www.dhsprogram.com) in STATA format.

### Key variables

The utilisation of postpartum family planning served as the outcome variable for the first and second goals (PPFP). This was defined as using a contraceptive method, whether modern or traditional, within a year of the birth of the most recent child. The survey's calendar data were used to create this variable. The woman's most recent birth date and the day she first began using a modern spacing method were used to calculate the number of months between the woman's most recent birth and the day she first started using a modern spacing method. This duration was modeled in the survival analysis.

The key explanatory variable was place of residence which is categorized into Rural, Urban Poor and Urban Non-Poor. Based on the literature review the variables were divided into following categories [37].

Under community level variables, geographical region and whether distance to health facility is a problem in community were included. Household level variables include sex of the household head, religion, and caste of head of the household.

The individual level variables include mother’s age at the time of birth, level of education of mother, parity, exposure to family planning messages through any media, mother’s correct knowledge of ovulatory cycle, ever termination of pregnancy, sex composition of living children,future desire for another children and union status.

The variables related to recent child birth include wanted status of last child, current breastfeeding status, sex of the recent child, utilization of Full antenatal care (ANC),post-natal care (PNC), Continuum of maternal health care services, health workers outreach for FP service. We also included PSUs level continuous variables. Those were ‘percentage of women having education secondary or higher’, ‘percentage of women from the rich and richest wealth quintile’ and ‘percentage of women who availed all the three MCH services.

### Analysis strategy

If a woman began utilising any method of family planning within a year of giving birth was the key outcome of interest. Cross tabulation and descriptive analysis were carried out for this. Another result was the length of time she started doing so. Survival analysis was deemed appropriate as a result. In this case, the “event” (failure) was the usage of postpartum family planning, and the “duration” was the number of months the woman used contraception after giving birth. All women who did not begin using contraception within 12 months were regarded to be survivors and were not followed up on, so it is unknown if they used any modern methods moving forward. The duration variable was measured up to 12 months (follow-up period). All women who did not begin using contraception within 12 months were deemed to be survivors and were not followed up on, so it is unknown if they used any modern methods moving ahead. The duration variable was measured up to 12 months (follow-up period). Women who had their last child less than a year ago and hadn't begun utilising a modern technique of family planning by the time of the survey were regarded as censored cases. A discrete-time hazards model was used to explicitly model the time of use.

### Multilevel Discrete-Time hazard model

It is assumed that time can take on only positive integer values (t = 1, 2, 3,...) and that we observe a total of n individuals (i = 1,..., n) nested under m primary sampling units (PSUs) (j = 1,..., m) beginning at some natural starting point t = 1. The observation continues until time${t}_{i}$, at which point either an event occurs or the observation is censored. Also observed is a K × 1 vector of explanatory variables${\mathbf{X}}_{it}$, which may take on different values at different discrete times.

The discrete-time hazard rate ${P}_{it}$ is the conditional probability that i^th^ individual experiencing event during interval *t*, given no earlier occurrence:

1 $${P}_{it} = Pr\left({T}_{i} = t\right|{T}_{i}\ge t, {\mathbf{X}}_{it}),$$

where T is the discrete random variable giving the uncensored time of event occurrence.

The next step is to specify how this hazard rate depends on time and the explanatory variables.

2 $${P}_{it} = 1 -exp[-exp({\alpha }_{t} + {\varvec{\beta}}\mathrm{^{\prime}}{\mathbf{X}}_{it})]$$

where ${{\varvec{\beta}}}^{^{\prime}}\mathrm{ is\;the\;coefficient\;vector}$ in the proportional hazards model of continuous-time scale. Note that ${\alpha }_{t}$ (t = 1, 2,...) is some function of time to model the baseline hazards function.

Equation (2) may be solved to yield the so-called complementary log–log function

3 $$log\left[-log\left(1 -{P}_{ijt}\right)\right]= {\alpha }_{t} + {{\varvec{\beta}}}^{^{\prime}}{\mathbf{X}}_{ijt}+{u}_{j}$$

Usually assume ${u}_{j} \sim N(0, {\sigma }_{u}^{2}$) where ${\sigma }_{u}^{2}$ represents unobserved heterogeneity or frailty.In our study,${P}_{ijt}$ is the probability that woman i in PSU j uses modern spacing method of contraception during interval *t*, given no earlier occurrence. This model is an example of what is called a two-level model—individual women (level 1) are nested within PSU (level 2). The purpose of this approach is to control for the correlation between women in a particular PSU. The PSU error term (${u}_{j}$) in the model gives an indication of the variation after controlling for the individual level characteristics.

## Results

### Results of urban poor definition

Results of Table 1 shows that estimates of urban poor population from Planning Commission estimates (2013) following Tendulkar methodology and based on NFHS-4 National wealth quintile (2015–16) (considering lowest two quintiles in urban areas considered as urban poor) are quite close. Since there is a gap of around 3 years between these two estimates, the proportion of rural population in each state should be either decreasing or stabilized from 2012 estimates and 2015–16 estimates. Only Kerala is showing increasing trend. Differences in Goa, Himanchal Pradesh and West Bengal were very smaller so they can be ignored.

**Table 1:** Comparison of population composition of India from planning commission estimates (2013) and National Family Health Survey-4 (2015–16)

**State**	**Planning commission estimates from Tendulkar methodology (Based on extrapolated population on 01/03/2012)**				**Based on NFHS-4 National wealth quintile (2015–16)** [lowest two quintiles in urban areas considered as urban poor]			
	**Urban Poor**	**Urban Non poor**	**Rural**	**Total**	**Urban Poor**	**Urban Non poor**	**Rural**	**Total**
Andhra Pradesh^a^	1.98	32.15	65.86	100	1.80	37.18	61.02	100
Arunachal Pradesh	4.66	18.26	77.08	100	1.86	24.63	73.51	100
Assam	2.91	11.29	85.80	100	2.32	12.67	85.01	100
Bihar	3.56	7.83	88.61	100	3.71	9.73	86.56	100
Chhattisgarh	5.84	17.74	76.42	100	3.22	21.54	75.24	100
Goa	2.58	60.52	36.90	100	1.77	61.09	37.14	100
Gujarat	4.37	38.74	56.89	100	2.12	42.3	55.57	100
Haryana	3.64	31.79	64.57	100	1.4	38.35	60.25	100
Himachal Pradesh	0.43	9.56	90.00	100	0.18	9.45	90.37	100
Jammu and Kashmir	1.97	25.45	72.57	100	1.8	28.09	70.11	100
Jharkhand	6.02	18.22	75.77	100	5.51	21.98	72.51	100
Karnataka	5.95	33.09	60.95	100	2.75	40.82	56.42	100
Kerala	2.49	47.63	49.87	100	0.51	46.37	53.12	100
Madhya Pradesh	5.83	21.92	72.25	100	3.84	26.9	69.26	100
Maharashtra	4.15	41.36	54.48	100	3.04	46.59	50.37	100
Manipur	10.02	20.74	69.24	100	9.36	31.18	59.46	100
Meghalaya	1.87	18.36	79.76	100	2.03	21.21	76.76	100
Mizoram	3.30	48.60	48.10	100	1.22	58.99	39.8	100
Nagaland	5.02	25.45	69.53	100	4.91	34.21	60.89	100
Delhi	9.62	88.12	2.26	100	1.51	97.7	0.79	100
Odisha	2.91	13.94	83.14	100	4.32	13.25	82.43	100
Puducherry	4.31	64.03	31.66	100	4.59	65.28	30.13	100
Punjab	3.50	34.38	62.12	100	0.65	38.96	60.39	100
Rajasthan	2.68	22.36	74.96	100	1.85	23.89	74.27	100
Sikkim	0.97	25.44	73.59	100	0.53	32.15	67.32	100
Tamil Nadu	3.20	45.69	51.12	100	3.53	47.36	49.11	100
Tripura	2.01	25.11	72.88	100	6.43	23.62	69.94	100
Uttar Pradesh	5.85	16.59	77.57	100	2.83	23.54	73.63	100
Uttarakhand	3.25	27.79	68.95	100	1.34	35.09	63.57	100
West Bengal	4.73	27.56	67.70	100	7.06	25.17	67.77	100
**Total**	**4.32**	**27.19**	**68.49**	**100**	**3.14**	**31.49**	**65.37**	**100**

Estimates of UTs are not shown in the table

^a^Estimate of Telangana and Andhra Pradesh are combined since poverty estimates were given only for Andhra Pradesh

We cannot say about the direction of increase or decrease in urban poor proportion between 2012 estimates and 2015–16 estimates. It can be hypothesized that Proportion of urban poor in each state should decrease over time period however, there can be rural to urban migration that can lead to increase in proportion of urban poor in some states. Therefore, it can be inferred from the table that use of National wealth quintile for classifying urban poor is justifiable.

### Results of descriptive analysis

Table 2 showing Distribution of first event occurred during 12 months of postpartum period after recent birth. Around 54.4 percent women did not start any method of contraception during 12 months of postpartum period in which 2.3 and 0.3 percent women experienced pregnancy and termination respectively in this duration. 8.3 percent women started use of traditional methods and 37.3 percent women started modern methods of contraception. In contrast to this, as per report of NFHS-4, overall 53.5 percent women adopted any method of contraception. Further, around 47.8 percent women were using modern methods of contraception.

**Table 2:** Distribution of first event occurred during 12 months of postpartum period after recent birth, India, NFHS-4

	**Not using**			**using**			**Frequency (weighed)**
**Time since last birth to contraceptive adoption**	**Not using**	**Pregnant**	**Termination**	**Traditional method**	**Modern method**	**Total Percent**	
1–2	-	0.09	0.01	2.10	16.30	18.50	25,336
3–4	-	0.15	0.02	2.47	7.52	10.16	13,914
5–6	-	0.31	0.05	1.65	5.47	7.48	10,248
7–8	-	0.35	0.05	1.13	3.93	5.46	7476
9–10	-	0.60	0.08	0.50	2.21	3.40	4657
11–12	-	0.79	0.07	0.45	1.86	3.18	4352
Not started yet	51.82	-	-	-	-	51.82	70,971
Total	51.82	2.29	0.28	8.32	37.30	100	136,953

Those who started contraception in 12-month postpartum period, mostly (40.3 percent) started within 2 months, 21.9 percent in 3^rd^-4^th^ month, 15.6 percent in 5^th^-6^th^ month, 11.1 percent in 7^th^-8^th^ month, 6 percent in 9^th^-10^th^ month and remaining 5.1 percent in last two months of postpartum period.

Those who experience pregnancy during 12-month postpartum period without start of any contraceptive (weighted *n* = 3131), more than one third of pregnancy occurred after 9^th^ month of postpartum period. It is worth to mention that around 10 percent of pregnancies occurred within 4 months of postpartum period.

There were 381 women (weighted) who did not start any contraception and experienced the abortion in the first month of pregnancy (Table 2).

However, when we analyzed deeply, there were some pregnancies and abortions which occurred after start of contraceptives (Table 3). Total 4046 women experienced pregnancy during 12-month postpartum period of which 3131 women did not start any contraceptives. More than 45 percent pregnancies resulted into abortion of which around 13 percent occurred into first month of pregnancy.

**Table 3:** Distribution of pregnancies that resulted into miscarriages, India, NFHS-4

Pregnancy status	Experienced Abortion		No Abortion	Total
	**First month**	**Rest months**		
**Pregnant**	518 (12.8%)	1314 (32.5%)	2214 (54.7%)	4046 (100%)
**Not Pregnant**	-	-	132,907 (100%)	132,907 (100%)
**Total**	518	1314	135,121	136,953

**Fig. 4:**
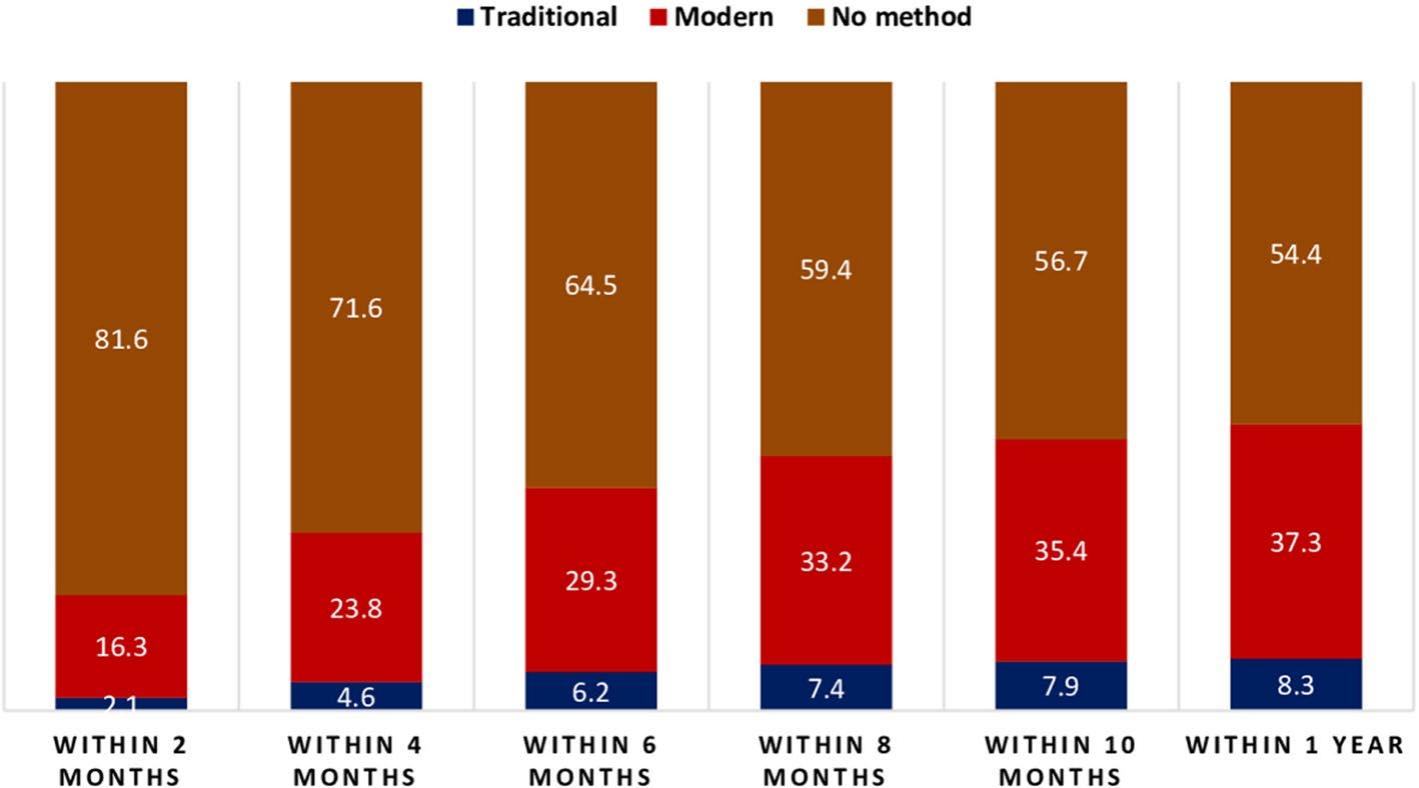
Distribution of postpartum contraceptive use over months, India, NFHS-4

Figure 4 showing month wise distribution of postpartum contraceptive use. More than 18 percent women started contraceptives within two months of the birth, of which 16 percent started modern method of contraception. Moreover, nearly 36 percent women started contraceptives within six months of the birth, of which 29 percent adopted modern contraceptives. At the end of postpartum period, more than 45 percent women adopted any method of contraception, of which approximately 37 percent women adopted modern contraceptives.

**Fig.5:**
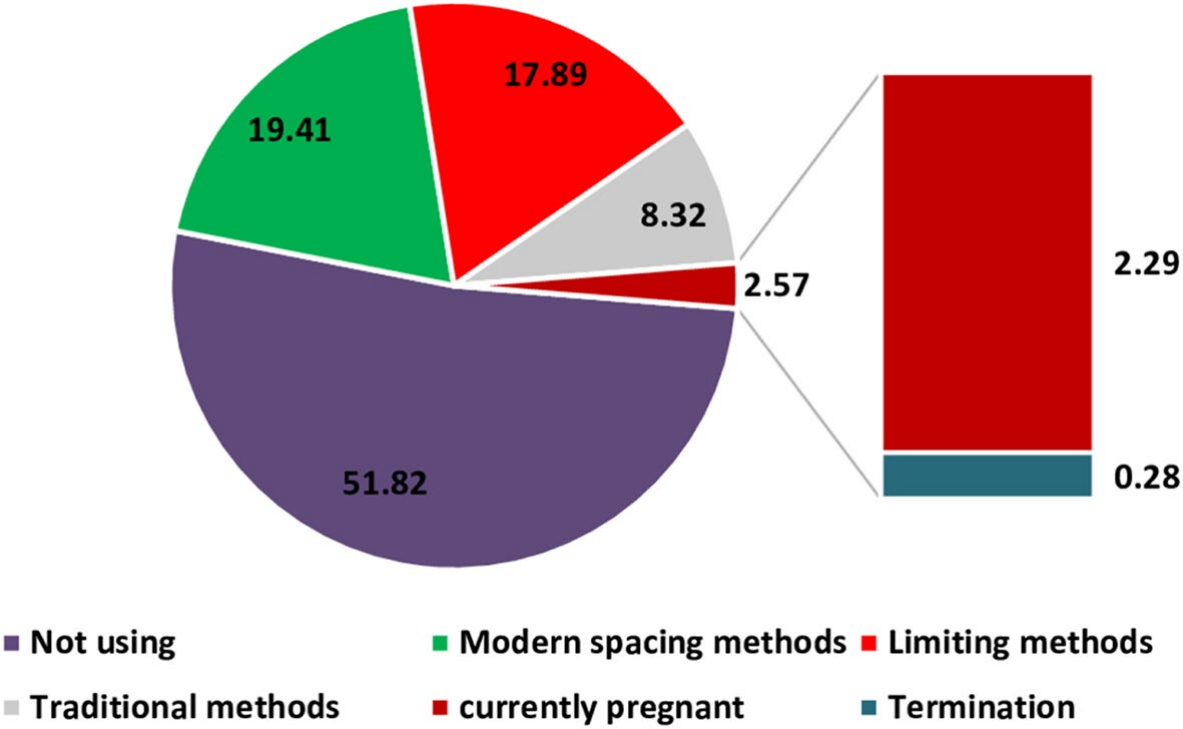
Distribution of postpartum contraceptive use over months, India, NFHS-4

By examining Fig. 5, it has been found that at the end of 12 months postpartum period, around 19 percent women started using modern spacing methods followed by 18 percent women choose limiting methods of contraception whereas 8 percent women started traditional methods. Around 3 percent women were those who did not initiate any method of contraception and were either pregnant or experienced abortion in first month of pregnancy.

For examining the pattern of contraceptive use during postpartum period, a comparison has been made among those women who adopted spacing, limiting and traditional methods at different months of postpartum periods (Table 4). Among women who had experienced any event (contraceptive/pregnancy/termination) within 2 months after delivery, a majority of the women or their partners (64%) had adopted limiting methods followed by the spacing methods (25%).This clearly shows that immediately after delivery, most of the couples were interested in adopting an effective contraceptive method.

**Table 4:** Percentage of contraceptive use and pregnancy outcome after having recent birth, India, NFHS-4

Time since last birth to contraceptive adoption	Type of contraceptive used and pregnancy outcome after having recent birth					
	**Modern spacing methods**	**Limiting methods**	**Traditional methods**	**currently pregnant**	**Termination**	**N Frequency (weighed)**
1–2	24.59	63.54	11.36	0.47	0.04	25,336
3–4	52.13	21.86	24.36	1.43	0.22	13,914
5–6	53.92	19.14	22.08	4.14	0.72	10,248
7–8	53.76	18.27	20.66	6.39	0.91	7,476
9–10	43.34	21.79	14.82	17.73	2.32	4,657
11–12	35.20	23.48	14.32	24.92	2.08	4,351
Total	40.28	37.14	17.26	4.75	0.58	65,982

Those women who had experienced any event (contraceptive/ pregnancy/ termination) between 3^rd^ to 8^th^ months, in each case, more than half women adopted spacing methods. Table 4 clearly shows that at the end of 12-month postpartum period, one in every fourth women reported that they were currently pregnant.

### Results of survival analysis

**Fig. 6:**
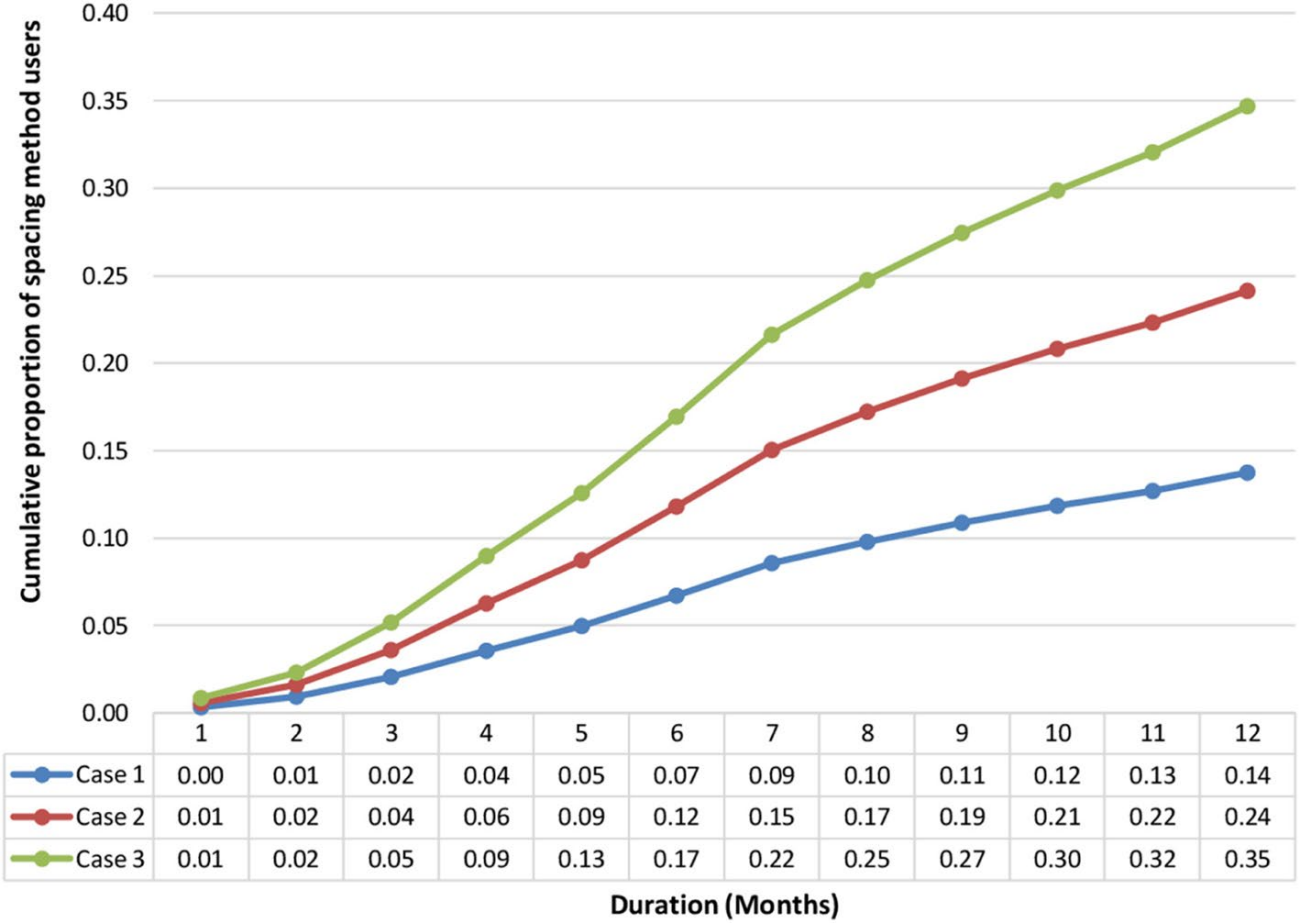
Cumulative proportion of women who started using any spacing method of family planning by duration from last childbirth, India, NFHS-4

Figure 6 depicts the proportion of spacing method users over time based on the reproductive calendar data for each of the three hypothetical considered cases. In Case 1, we assumed that all the non-users of contraceptives are assumed to start modern spacing contraceptives after 12 months follow up. In Case 2, we assumed that 50 percent of the non-users of contraceptives are assumed to start modern spacing contraceptives after 12 months follow up. In Case 3, we assumed that 30 percent of the non-users of contraceptives are assumed to start modern spacing contraceptives after 12 months follow up.

Five, four and two percent of women had started using spacingcontraceptives by the end of the 3rd month after childbirth in Case 3, 2 and 1 respectively. In each case, the proportion of users shows agradual increase in the level of spacing contraceptive use over the months. In Case 1, the proportion reach 7% after 6 months, 11% after 9 months, and 14% after 12 months. In Case 2, the proportion reach 12% after 6 months, 19% after 9 months, and 24% after 12 months. In Case 3, the proportion reach 17% after 6 months, 27% after 9 months, and 35% after 12 months.

Moreover, the Log-Rank test was performed to examine the significance of the differences among the survival curves for various categories of each of the background characteristics. Table 5 summarizes the results for Case 1, 2 and 3.

**Table 5:** Results of Log-Rank test for equality of survivor functions of postpartum family planning use for selected background characteristics, India, NFHS-4

Background characteristics	Degrees of freedom (df)	Case 1 (*N* = 94,272)		Case 2 (*N* = 60,196)		Case 3 (*N* = 46,565)	
		**Chi square**	***p*-value**	**Chi square**	***p*-value**	**Chi square**	***p*-value**
Residence	2	8457.99	*p* < 0.001	7506.51	*p* < 0.001	7088.43	*p* < 0.001
Mother's age at birth	3	854.09	*p* < 0.001	743.37	*p* < 0.001	625.86	*p* < 0.001
Level of education	3	15,412.81	*p* < 0.001	13,693.32	*p* < 0.001	11,905.21	*p* < 0.001
Religion	2	2355.6	*p* < 0.001	1889.7	*p* < 0.001	1840.39	*p* < 0.001
Caste	3	9598.23	*p* < 0.001	8040.49	*p* < 0.001	6982.11	*p* < 0.001
Region	5	29,115.53	*p* < 0.001	25,498.79	*p* < 0.001	21,243.46	*p* < 0.001
Sex of head of household	1	1631.13	*p* < 0.001	1428.3	*p* < 0.001	1183.55	*p* < 0.001
Media exposure to FP message	1	12,632.13	*p* < 0.001	11,324.69	*p* < 0.001	10,041.82	*p* < 0.001
Correct knowledge of ovulatory cycle	1	11,261.52	*p* < 0.001	9502.53	*p* < 0.001	8643.97	*p* < 0.001
Ever termination of pregnancy	1	155.87	*p* < 0.001	111.79	*p* < 0.001	141.17	*p* < 0.001
Sex composition of children	5	3383.83	*p* < 0.001	2937.19	*p* < 0.001	2668.79	*p* < 0.001
Wanted last child	2	783.44	*p* < 0.001	751.46	*p* < 0.001	579.05	*p* < 0.001
Breastfeeding status at survey date	1	67.49	*p* < 0.001	61.79	*p* < 0.001	37.53	*p* < 0.001
Future desire for next children	2	5881.4	*p* < 0.001	5409.53	*p* < 0.001	4904.31	*p* < 0.001
Health outreach	2	8930.92	*p* < 0.001	7873.23	*p* < 0.001	6402.31	*p* < 0.001
Full ANC	1	5679.26	*p* < 0.001	5008.49	*p* < 0.001	3695.68	*p* < 0.001
Delivery by skilled birth attendant	1	4039.96	*p* < 0.001	3778.39	*p* < 0.001	3308.63	p < 0.001
PNC	1	9541.29	*p* < 0.001	8907.06	*p* < 0.001	6803.38	*p* < 0.001
Continuum of maternal and child health care services	3	12,816.55	*p* < 0.001	11,725.19	*p* < 0.001	9214.83	*p* < 0.001
Whether distance to health facility is a problem	2	4826.01	*p* < 0.001	4431.94	*p* < 0.001	4022.03	*p* < 0.001
Parity	1	964.03	*p* < 0.001	831.45	*p* < 0.001	628.79	*p* < 0.001
Whether delivery by C-section	1	2625.66	*p* < 0.001	2200.42	*p* < 0.001	1942.12	*p* < 0.001
Sex of recent birth	1	61.94	*p* < 0.001	35.03	*p* < 0.001	95.4	*p* < 0.001
Whether recent child is alive	1	1285.32	*p* < 0.001	1181.65	*p* < 0.001	965.96	*p* < 0.001

### Results of Multilevel Discrete-Time hazard models

The present objective was undertaken to test whether there is a difference in propensity to adopt postpartum contraceptives between women belongs to rural and urban poor. Another objective is to test whether the difference persists after adjusting for utilization of MCH services and other selected background characteristics. For previous objectives, we were focusing on adoption of any contraceptives (spacing, limiting or traditional methods), but at the time of discrete-time complementary log–log multilevel modelling, we have excluded those women who were using traditional or sterilization method. The probable reason for exclusion could be because medical professionals educate couples about using modern contraceptives early to lengthen the period between pregnancies at the time that MCH services are used. As a result, we have omitted women who started using traditional methods of contraception after giving birth. Women and their husbands were also omitted from the analysis if they had sterilisation following their most recent childbirth. The dependent variable was time to begin using any modern spacing techniques versus no techniques.

Table 6 presents the results obtained from the discrete-time complementary log–log multilevel model on adoption of modern spacing methods in India during 2015–16. We presented the results for each of the three considered cases defined in previous section. Model 1 & Model 2 were presented for the Case 1 (when all the non-users at the end of follow up period were assumed to start spacing method after 12 months). Similarly, Model 3 & Model 4 were for the Case 2 (where 50 percent of the non-users at the end of follow up period were assumed to start spacing method after 12 months) and Model 5 & Model 6 were for the Case 3 (where only 30 percent of the non-users at the end of follow up period were assumed to start spacing method after 12 months). Model 1, Model 3 and Model 5, only residence and month of contraceptive adoption (duration variable) were included. Whereas, Model 2, Model 4 and Model 6 were adjusted for utilization of MCH services as well as other selected background characteristics.

**Table 6:** Results obtained from discrete-time complementary log–log multilevel models on spacing contraceptives adoption by selected background characteristics, India, NFHS-4

Background Characteristics	Case 1		Case 2		Case 3	
	**Model 1**	**Model 2**^**a**^	**Model 3**	**Model 4**^**a**^	**Model 5**	**Model 6**^**a**^
	**Hazard ratio [95% CI]**	**Hazard ratio [95% CI]**	**Hazard ratio [95% CI]**	**Hazard ratio [95% CI]**	**Hazard ratio [95% CI]**	**Hazard ratio [95% CI]**
**Month of contraceptive adoption**						
1–2 (Ref.)	1	1	1	1	1	1
3–4	1.531*** [1.443,1.624]	1.531*** [1.444,1.624]	1.682*** [1.583,1.788]	1.661*** [1.564,1.764]	1.796*** [1.690,1.909]	1.755*** [1.652,1.863]
5–6	1.515*** [1.417,1.619]	1.541*** [1.442,1.646]	1.831*** [1.708,1.963]	1.833*** [1.712,1.964]	2.107*** [1.963,2.262]	2.075*** [1.935,2.226]
7–8	1.370*** [1.266,1.482]	1.419*** [1.312,1.535]	1.829*** [1.682,1.989]	1.870*** [1.721,2.032]	2.306*** [2.115,2.514]	2.315*** [2.125,2.521]
9–10	0.775*** [0.704,0.854]	0.812*** [0.738,0.894]	1.109* [1.001,1.227]	1.151** [1.041,1.273]	1.492*** [1.344,1.657]	1.524*** [1.374,1.691]
11–12	0.634*** [0.569,0.708]	0.668*** [0.598,0.746]	0.950 [0.849,1.064]	0.995 [0.888,1.114]	1.342*** [1.197,1.505]	1.386*** [1.235,1.555]
**Residence**						
Rural (Ref.)	1	1	1	1	1	1
Urban poor	1.267** [1.096,1.465]	1.120 [0.975,1.285]	1.284*** [1.110,1.486]	1.154* [1.011,1.318]	1.281*** [1.106,1.484]	1.146* [1.006,1.306]
Urban non-poor	1.906*** [1.809,2.008]	1.175*** [1.106,1.248]	1.873*** [1.778,1.974]	1.182*** [1.113,1.255]	1.854*** [1.760,1.953]	1.208*** [1.137,1.283]
**Mother’s age at birth**						
30 + (Ref.)		1		1		1
Below 18		1.232** [1.053,1.442]		1.347*** [1.154,1.574]		1.159 [0.980,1.371]
18–24		1.194*** [1.098,1.299]		1.236*** [1.136,1.345]		1.182*** [1.080,1.295]
25–30		1.140** [1.050,1.239]		1.165*** [1.069,1.270]		1.119* [1.020,1.228]
**Level of education**						
No education (Ref.)		1		1		1
Primary		1.266*** [1.167,1.374]		1.229*** [1.133,1.333]		1.187*** [1.088,1.294]
Secondary		1.451*** [1.334,1.579]		1.382*** [1.265,1.510]		1.323*** [1.207,1.449]
Higher		1.774*** [1.600,1.966]		1.663*** [1.493,1.853]		1.468*** [1.312,1.643]
**Religion**						
Hindu (Ref.)		1		1		1
Muslim		1.358*** [1.266,1.457]		1.301*** [1.213,1.396]		1.322***[1.237,1.412]
Others		1.047 [0.956,1.147]		1.023 [0.938,1.115]		1.013 [0.933,1.101]
**Caste**						
Schedule Tribe (Ref.)		1		1		1
Schedule Caste		1.380*** [1.233,1.545]		1.333*** [1.187,1.497]		1.346*** [1.178,1.537]
Other Backward Caste		1.177** [1.066,1.299]		1.149** [1.040,1.269]		1.180** [1.053,1.322]
General		1.341*** [1.206,1.490]		1.304*** [1.173,1.450]		1.331*** [1.181,1.500]
**Region**						
East (Ref.)		1		1		1
North zone		1.509*** [1.407,1.619]		1.468*** [1.370,1.573]		1.376*** [1.284,1.476]
West zone		0.588*** [0.534,0.647]		0.637*** [0.580,0.701]		0.686*** [0.625,0.753]
South zone		0.168*** [0.150,0.188]		0.214*** [0.191,0.240]		0.270*** [0.239,0.305]
Central zone		0.897** [0.837,0.961]		0.965 [0.902,1.034]		0.967 [0.903,1.036]
Northeast zone		1.524*** [1.404,1.654]		1.459*** [1.349,1.579]		1.330*** [1.231,1.437]
**Sex of head of household**						
Female (Ref.)		1		1		1
Male		1.310*** [1.214,1.415]		1.260*** [1.168,1.360]		1.256*** [1.167,1.352]
**Media exposure to FP message**						
No (Ref.)		1		1		1
Yes		1.281*** [1.199,1.369]		1.269*** [1.188,1.355]		1.259*** [1.175,1.349]
**Correct knowledge of ovulatory cycle**						
No (Ref.)		1		1		1
Yes		1.253*** [1.186,1.324]		1.232*** [1.163,1.305]		1.223*** [1.155,1.294]
**Ever termination of pregnancy**						
Yes (Ref.)		1		1		1
No		1.195*** [1.124,1.271]		1.122*** [1.055,1.192]		1.070* [1.009,1.135]
**Sex composition of children**						
No children (Ref.)		1		1		1
No sons but at least one daughter		4.022*** [2.785,5.809]		3.187*** [2.201,4.615]		2.967*** [2.005,4.389]
No daughters but at least one son		4.370*** [3.025,6.312]		3.337*** [2.307,4.827]		3.131*** [2.116,4.633]
Sons less than daughters		4.728*** [3.255,6.866]		3.625*** [2.487,5.284]		3.118*** [2.092,4.646]
Sons greater than daughters		4.477*** [3.072,6.527]		3.549*** [2.427,5.188]		3.174*** [2.124,4.745]
Equal sons and daughters		4.678*** [3.234,6.767]		3.573*** [2.462,5.186]		3.286*** [2.214,4.876]
**Wanted last child**						
Wanted then		1		1		1
Wanted later (Mistimed)		1.296*** [1.166,1.439]		1.304*** [1.176,1.446]		1.179** [1.061,1.310]
Wanted no more (Unwanted)		1.039 [0.916,1.178]		1.116 [0.989,1.259]		1.093 [0.958,1.247]
**Future desire for next children**						
Wanted more (Ref.)		1		1		1
Wants no more		1.182*** [1.119,1.248]		1.186*** [1.122,1.255]		1.186*** [1.120,1.256]
Sterilized/Infecund/Never had sex		0.649*** [0.581,0.726]		0.691*** [0.616,0.775]		0.742*** [0.665,0.828]
**Health worker’s outreach for FP service**						
Not met (Ref.)		1		1		1
Met but not received advice on family planning		1.066 [0.993,1.143]		1.063 [0.991,1.141]		1.037 [0.966,1.114]
Met and received advice on family planning		1.431*** [1.353,1.514]		1.395*** [1.317,1.478]		1.336*** [1.259,1.418]
**Continuum of maternal and child health care services**						
No service (Ref.)		1		1		1
Any one service		1.148** [1.041,1.267]		1.123* [1.022,1.234]		1.079 [0.971,1.199]
Any two services		1.387*** [1.263,1.524]		1.344*** [1.233,1.467]		1.255*** [1.138,1.384]
All the three services		1.573*** [1.389,1.782]		1.562*** [1.393,1.751]		1.377*** [1.213,1.564]
**Whether distance to health facility is a problem**						
No problem (Ref.)		1		1		1
Big problem		0.916** [0.860,0.976]		0.932* [0.877,0.990]		0.941 [0.883,1.003]
Not a big problem		0.941* [0.889,0.996]		0.957 [0.906,1.011]		0.979 [0.927,1.034]
Constant^b^	-4.399*** (0.0312)	-7.672*** (0.2080)	-3.899*** (0.0333)	-6.824*** (0.2080)	-3.570*** (0.0336)	-6.265*** (0.2190)
Var(constant)^b^	1.758*** (0.0435)	1.103*** (0.0343)	1.540*** (0.0459)	0.983*** (0.0371)	1.371*** (0.0470)	0.926*** (0.0403)
Total person-month (n)	948,046	948,046	539,134	539,134	375,562	375,562

^a^Model 2, Model 4 and Model 6 are also adjusted for status of breastfeeding at survey date and PSU level explanatory variables (PSU level percentage of women having education secondary or higher, PSU level percentage of women from the rich and richest wealth quintile, PSU level percentage of women who availed all the three MCH services)

^b^for constant and Var(constant) beta coefficients and their standard errors were reported in the parenthesis

95% confidence intervals are in square brackets

^*^ *p* < 0.05, ** *p* < 0.01, *** *p* < 0.001

Findings from the Model 1, Model 3 and Model 5 showed that, women from rural areas had a lower chance of early initiation of modern spacing methods after having recent birth as compare to that of Urban Poor and Urban non-poor areas. However, when we adjusted the results for utilization of MCH services and selected background characteristics, coefficient for Urban Poor under Model 2 became insignificant. On the other hand, under Model 4, Model 6, the hazard ratio for the women of both Urban Poor and Urban non-poor areas with respect to that of Rural areas were lower in comparison to corresponding hazard ratio obtained from Model 3 and Model 5.

Women from North and Northeast regions had higher chance of early adoption of spacing contraception than that of East region. Moreover, the absence of early initiation of modern spacing methods was also found among women from west, south and central regions of India and for those women who considered that distance between health facility and their home was a big problem. Further, parity, delivery by C-section (results were not presented for parity and delivery by C-section), other religion, missed opportunity of health worker regarding FP information and unwanted recent birth were not associated with early adoption of modern contraceptive after controlling for socioeconomic characteristics of women and utilization of MCH services.

As expected, Women’s education was positively related to the early initiation of modern spacing methods whereas women’s age was negatively linked to the early initiation of modern spacing methods. It is worth to mention that chances of early adoption of spacing contraception was high among Muslim women. In comparison to women of scheduled tribe caste, women from scheduled caste and General caste communities had higher chance of early spacing contraceptive adoption. It has been found that women from male headed household, correct knowledge of ovulatory cycle and exposure to media were positively associated with the early initiation of modern spacing methods. Moreover, women who ever experienced termination or miscarriage had lower chance of early contraceptive adoption.

The results also reflect that sex composition of children born was associated with the early initiation of spacing contraceptives. Women having no son in their family had lower chances of early initiation of spacing contraception as compare to that have no daughter in the family. Women whose last birth was mistimed (wanted later) had higher chance of early adoption of contraception than women whose last birth was wanted. Furthermore, women having more desire for another child had lesser chance of early adoption of spacing contraceptives as compare to that having no more desire for next child.

Women who met health workers and received advice on family planning had higher chance of early initiation of spacing contraception as compare to rest women. As expected, continuum of maternal and child health care services (full ANC, delivery by skilled health personal and PNC) was positively associated with the early initiation of spacing contraception during postpartum period. The women receiving all the MCH services mentioned above had higher chances to adopt contraception early than any other women. An explanation for this might be that at the time of utilization of MCH services, the health workers provide the information about the availability and accessibility of various contraceptive methods. Once women come to know about the different types of birth control methods, the chances are higher than they would adopt contraceptive methods.

### Predicted probabilities for adoption of spacing contraceptives

**Fig. 7:**
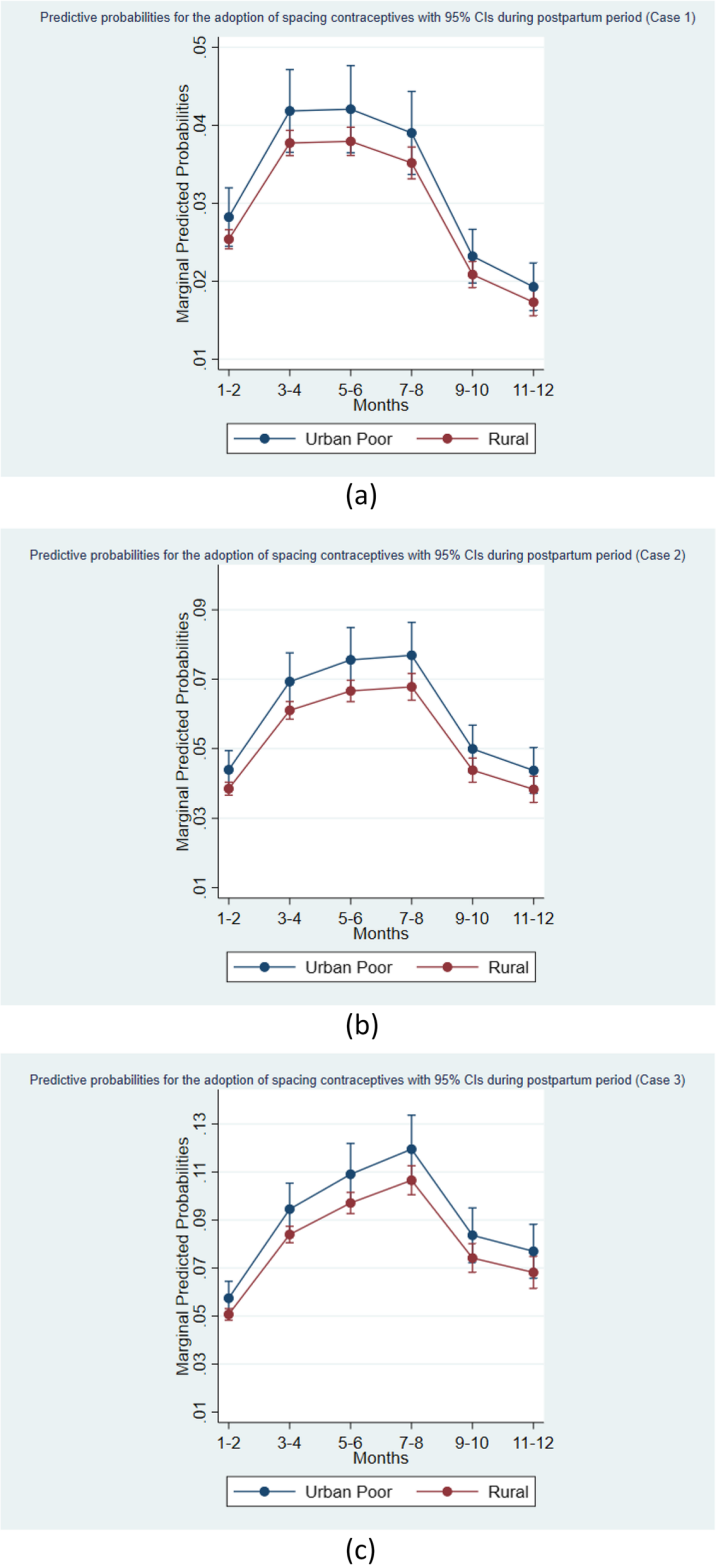
**a** predicted probabilities of adoption of spacing contraceptives with 95% CI during postpartum period (Case 1), (**b**) predicted probabilities of adoption of spacing contraceptives with 95% CI during postpartum period (Case 2), (**c**) predicted probabilities of adoption of spacing contraceptives with 95% CI during postpartum period (Case 3)

Figures 7 (a), (b) and (c) display the predicted probabilities of adoption of spacing contraceptives by a woman having particular characteristics, once other characteristics are retained at their average level in the respective. Cases 1, 2 and 3. In Case 1, the predicted probability of contraceptive adoption by a rural woman first increases from 0.025 (first two months) to 0.038 (in 5^th^-6^th^ month) and then decline to 0.017 in the last two months of the study period. On the other hand, the predicted probability of initiating spacing contraception by a urban poor women increases from 0.028 (in first two months) to 0.042 (in 5^th^-6^th^ month) and then decrease to 0.019 (in 11^th^-12^th^ month).

Moreover, under Case 2, the predicted probability for the contraceptive adoption by a rural woman increases from 0.038 (in first two months) to 0.068 (in 7^th^-8^th^ month) and then decline to 0.038 (in 11^th^-12^th^ month). While the corresponding predicted probability for women from urban poor area is 0.044 in the first two months, reaches to peak in the 7^th^-8^th^ month (0.077) and then decline to 0.044 in the last two months of the considered postpartum period.

Furthermore, under Case 3, the predicted probability of early initiation of spacing contraception by a rural woman starts from 0.051 (in first two months) increases to 0.11 (in 7^th^-8^th^ month) and then decline to 0.068 (in 11^th^ -12^th^ month). Whereas the corresponding predicted probability for women of urban poor area starts with 0.057 (in first two months), reaches to peak in 7^th^-8^th^ month (0.12) then decreases to 0.077 in the last two months of the study period.

It has been clear from the above description that from the predicted probabilities found under Case 1 are consistently lower than that obtained under Case 2 and Case 3. Similarly, the predicted probabilities obtained under Case 2 are consistently lower than that estimated under Case 3. In other words, we can say that as the number of censored cases come down, the predicted probabilities of spacing contraceptives adoption by a woman increases. In each of the Figs. 7(a), (b) and (c), the confidence intervals corresponding to their point estimates of predicted probabilities for rural and urban poor women are overlapping.

## Discussion and conclusion

Despite several policies and initiatives to provide access to family planning services, there is still a significant unmet need for family planning during the postpartum period in many developing nations, including India [38]. In the "Collective Action for Postpartum Family Planning" statement from the London Summit, the prevention of unwanted and closely spaced pregnancies within the first year following delivery is defined as postpartum family planning. It emphasises how crucial it is to stress counselling and offer postpartum mothers strategies.

The results clearly show that women from rural areas had a lower chance of early initiation of modern spacing methods after having a recent child as compared to those of urban poor and urban non-poor areas after accounting for the socioeconomic and demographic variables. The observed inequalities between rural and urban areas in the early adoption of spacing techniques may be explained by the disparities in the accessibility of social resources including education, information, and family planning services. Women did not early adopt spacing practises in the south and west, two areas with low fecundity. This could be a result of early nonreversible technique adoption being especially common among couples in these areas [39]. Interestingly, Muslim women were less likely than Hindu women to use postpartum contraception at the end of the 12-month postpartum period, but they were more likely to start using spacing contraception. There is a lot of scope for promoting the use of spacing methods because a prior study on NFHS-3 revealed that scheduled caste and scheduled tribe groups had a higher risk of delaying the use of modern contraceptives when compared to other castes [37]. Our findings, however, presented a different picture. Our results revealed that, overall, family planning initiation was found to be similar across women from scheduled caste and women from scheduled tribes compared to women from scheduled tribes, even though the overall prevalence of PPFP was lower among women from scheduled caste. The findings show that elderly women were less likely to start their contraceptives earlier than younger people. It was determined that the majority of younger women who were recently married were probably unaware of the appropriate timing for using contraceptives. On the other hand, younger women who were more likely to engage in frequent sexual activity required greater accessibility to spacing techniques to lower the risk of unintended or premature pregnancies. Older women were more likely to use the spacing strategy later than younger women, which may be related to their decreased sexual activity and decision to undergo sterilisation once they had the number of children they wanted. Similar findings have also been observed in other studies conducted in India [40]. The analysis also indicates that women with any level of education are less likely to delay starting contraception than illiterates. The educated women may be more efficient at communicating their desires for conception or may have received appropriate health education and services from professionals [41].

Women who have lost a child should give their bodies some time to heal before attempting conception again, however the results of the study suggest that these women were more likely to delay using modern contraception than the women who had not lost any children. This may be related to their wish to raise a child in place of a deceased one or to prevent childlessness. Early commencement of contraceptive use after childbirth is significantly positively influenced by exposure to electronic mass media, such as radio and television. Our result did not show any significant association between parity, breastfeeding status, mode of delivery (C-section) and early initiation of contraception. However our findings are in contrast to results of study [37], which shows that women who continued breastfeeding were less likely to early adopt contraceptives as compared with those currently breastfeeding.

The fact that the use of the MCH services that were taken into account in this study—ANC visits, skillful delivery, and postnatal care—was positively linked with contraceptive adoption during the postpartum period, even after controlling for other background variables, was a significant finding. This relationship matched the results that were previously reported [10, 42]. It’s possible that when women use MCH services, they interact with health professionals and learn the value of early contraceptive uptake and a healthy interpregnancy gap. The findings suggest that prenatal, postpartum, and antenatal care services provided crucial windows of opportunity for women to learn where and when to get contraception.

Despite the government initiative in India offering free maternal health and family planning services to users, many chances to access counselling and other family planning services are missed. Evidence of this includes a high degree of unmet family planning need and an almost constant level of family planning use. Additionally, it can be challenging to acquire services in the nation's rural and remote locations. 

The results of this study confirm the importance of education and counselling in encouraging women to use maternal health services continuously from the beginning of pregnancy to delivery. This is necessary for the country to support women in keeping in touch with medical professionals and ensuring their reproductive health. Instead than only concentrating on family planning, policy planners must concentrate on targeted interventions for use of family planning during the postpartum period. As part of the National Family Welfare Program, the Government of India has already implemented a number of novel initiatives, including Mission Parivar Vikas, home contraceptive delivery by ASHA staff, emphasis on Postpartum Family Planning (PPFP), awareness of the new and reversible contraceptive basket of choice, etc. However, in order to meet the Sustainable Development Goals’ (SDGs) target of meeting 75% of the global demand for modern contraceptives by 2030, it is vital to design an intervention that would result in efficient service delivery.  

## Data Availability

The dataset analyzed cannot be made publicly available by us as it belongs to the DHS program, but it can be accessed from the following link after acquiring permission from Measure DHS: https://www.dhsprogram.com/data/available-datasets.cfm.
